# Supplementary material for: A high-resolution mRNA expression time course of embryonic development in zebrafish
Source: eLife. 2017 Nov 16;6:e30860. doi: 10.7554/eLife.30860 (PMC5690287; doi:10.7554/eLife.30860)
Supplement: Supplementary file 6. [file elife-30860-supp6.zip › biolayout-clusters-files/Cluster147.html]

Cluster147


# Cluster147: Detail

### Go to ZFA detail

## GO

| GO ID | Description | Domain | Annotated | Expected | Observed | Adjusted p-value | Genes | Ensembl IDs |
| --- | --- | --- | --- | --- | --- | --- | --- | --- |
| GO:0007275 | multicellular organismal development | biological\_process | 1969 | 1.8 | 7 | 0.036 | hoxa11b fezf1 ngfb hoxa2b myod1 hoxa10b ccdc151 | ENSDARG00000007009 ENSDARG00000010270 ENSDARG00000014050 ENSDARG00000023031 ENSDARG00000030110 ENSDARG00000031337 ENSDARG00000062978 |
